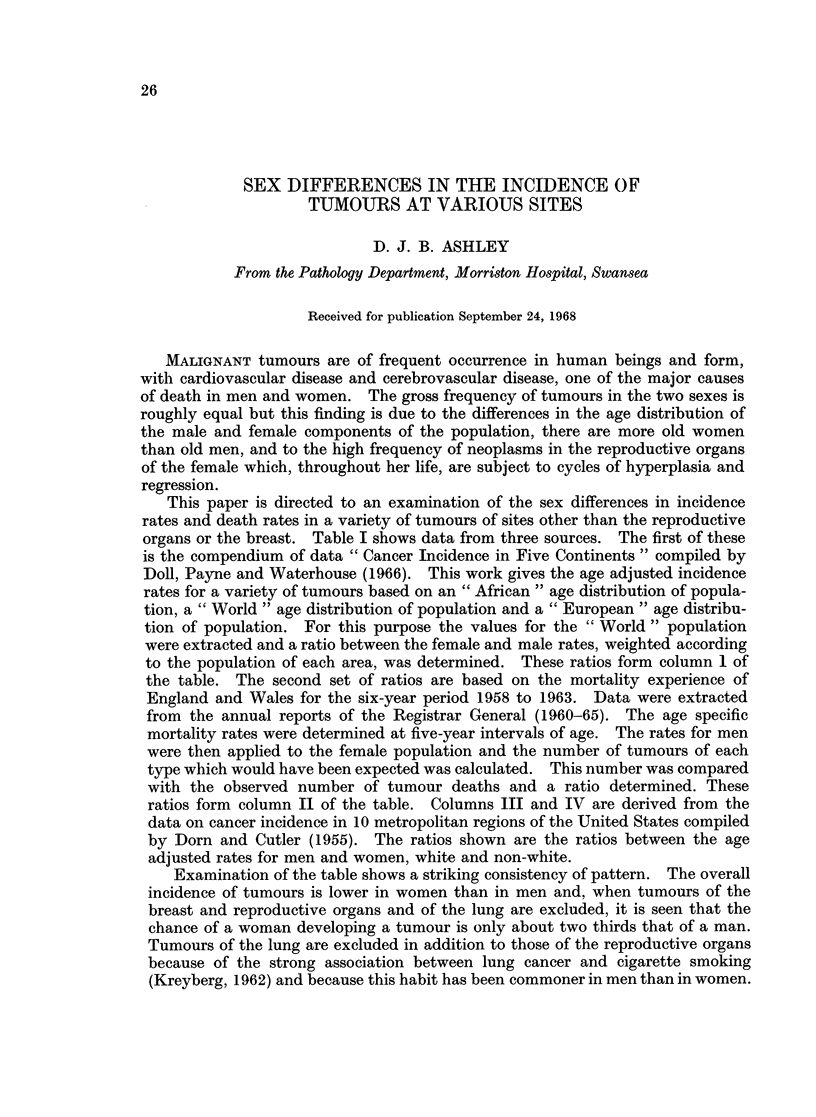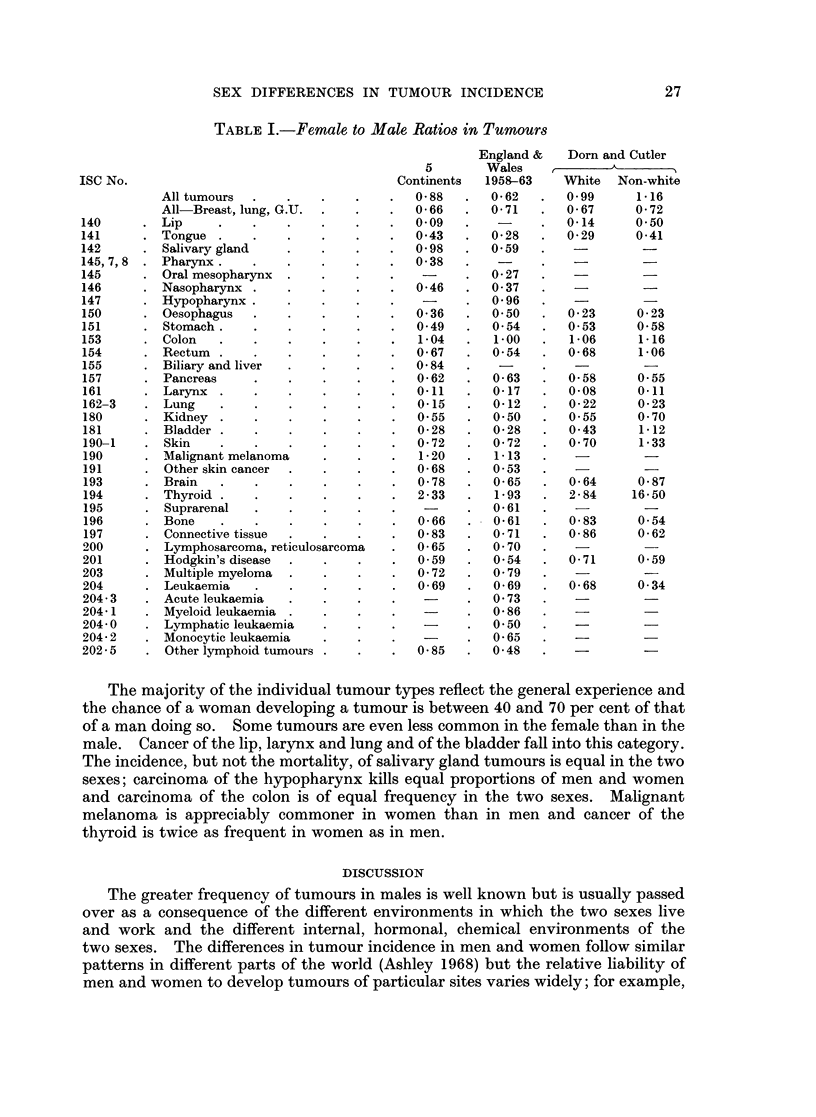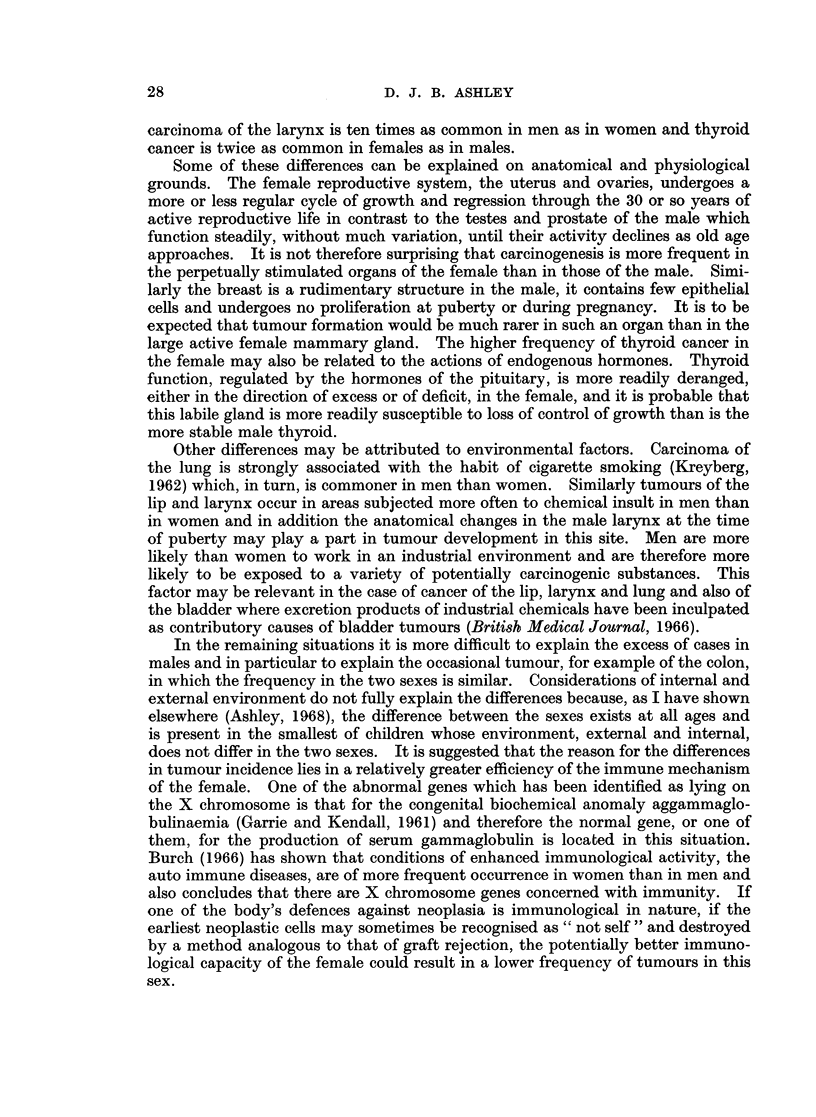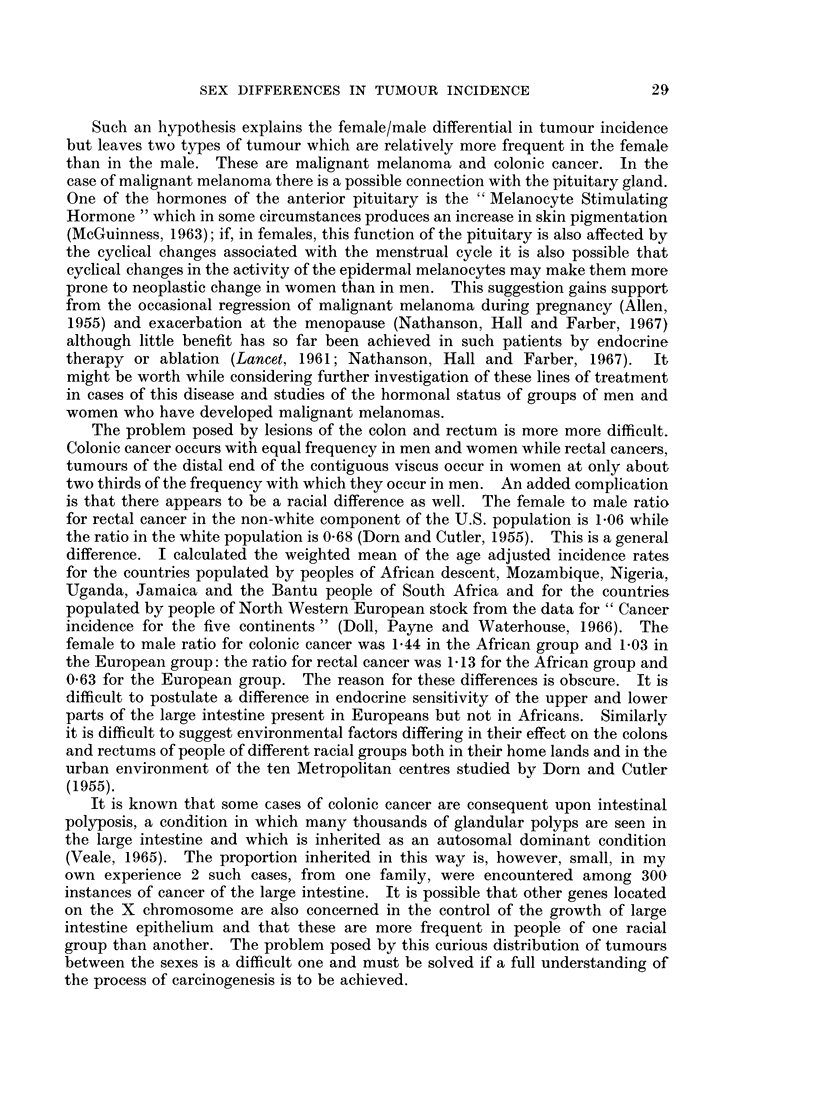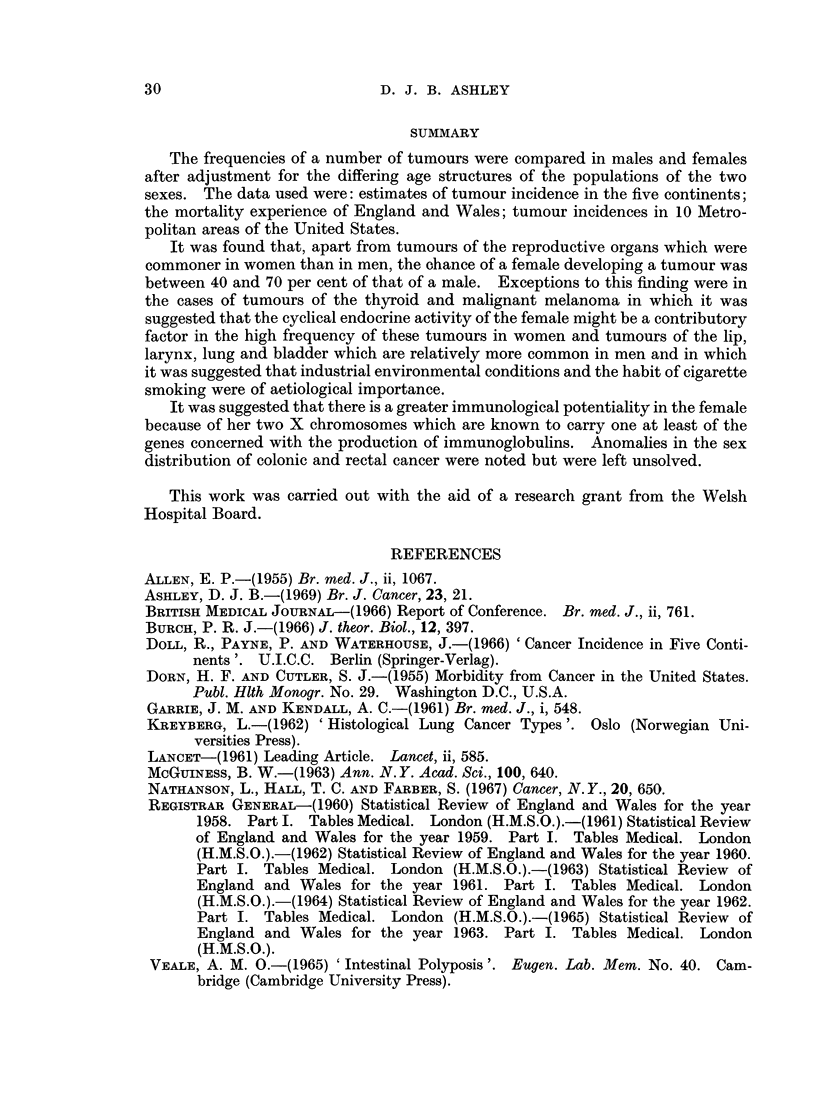# Sex differences in the incidence of tumours at various sites.

**DOI:** 10.1038/bjc.1969.4

**Published:** 1969-03

**Authors:** D. J. Ashley


### SEX DIFFERENCES IN THE INCIDENCE OF TUMOURS AT VARIOUS SITES

D. J. B. ASHLEY

*From the Pathology Department, Morriston Hospital, Swansea*

Received for publication September 24, 1968

MALIGNANT tumours are of frequent occurrence in human beings and form, with cardiovascular disease and cerebrovascular disease, one of the major causes of death in men and women. The gross frequency of tumours in the two sexes is roughly equal but this finding is due to the differences in the age distribution of the male and female components of the population, there are more old women than old men, and to the high frequency of neoplasms in the reproductive organs of the female which, throughout her life, are subject to cycles of hyperplasia and regression.

This paper is directed to an examination of the sex differences in incidence rates and death rates in a variety of tumours of sites other than the reproductive organs or the breast. Table I shows data from three sources. The first of these is the compendium of data " Cancer Incidence in Five Continents " compiled by Doll, Payne and Waterhouse (1966). This work gives the age adjusted incidence rates for a variety of tumours based on an " African " age distribution of population, a " World " age distribution of population and a " European " age distribution of population. For this purpose the values for the " World " population were extracted and a ratio between the female and male rates, weighted according to the population of each area, was determined. These ratios form column 1 of the table. The second set of ratios are based on the mortality experience of England and Wales for the six-year period 1958 to 1963. Data were extracted from the annual reports of the Registrar General (1960–65). The age specific mortality rates were determined at five-year intervals of age. The rates for men were then applied to the female population and the number of tumours of each type which would have been expected was calculated. This number was compared with the observed number of tumour deaths and a ratio determined. These ratios form column II of the table. Columns III and IV are derived from the data on cancer incidence in 10 metropolitan regions of the United States compiled by Dorn and Cutler (1955). The ratios shown are the ratios between the age adjusted rates for men and women, white and non-white.

Examination of the table shows a striking consistency of pattern. The overall incidence of tumours is lower in women than in men and, when tumours of the breast and reproductive organs and of the lung are excluded, it is seen that the chance of a woman developing a tumour is only about two thirds that of a man. Tumours of the lung are excluded in addition to those of the reproductive organs because of the strong association between lung cancer and cigarette smoking (Kreyberg, 1962) and because this habit has been commoner in men than in women.

TABLE I.—*Female to Male Ratios in Tumours*

| ISC No. | | 5 Continents | England & Wales 1958–63 | Dorn and Cutler White | Dorn and Cutler Non-white |
|---|---|---|---|---|---|
| | All tumours | 0·88 | 0·62 | 0·99 | 1·16 |
| | All—Breast, lung, G.U. | 0·66 | 0·71 | 0·67 | 0·72 |
| 140 | Lip | 0·09 | — | 0·14 | 0·50 |
| 141 | Tongue | 0·43 | 0·28 | 0·29 | 0·41 |
| 142 | Salivary gland | 0·98 | 0·59 | — | — |
| 145, 7, 8 | Pharynx | 0·38 | — | — | — |
| 145 | Oral mesopharynx | — | 0·27 | — | — |
| 146 | Nasopharynx | 0·46 | 0·37 | — | — |
| 147 | Hypopharynx | — | 0·96 | — | — |
| 150 | Oesophagus | 0·36 | 0·50 | 0·23 | 0·23 |
| 151 | Stomach | 0·49 | 0·54 | 0·53 | 0·58 |
| 153 | Colon | 1·04 | 1·00 | 1·06 | 1·16 |
| 154 | Rectum | 0·67 | 0·54 | 0·68 | 1·06 |
| 155 | Biliary and liver | 0·84 | — | — | — |
| 157 | Pancreas | 0·62 | 0·63 | 0·58 | 0·55 |
| 161 | Larynx | 0·11 | 0·17 | 0·08 | 0·11 |
| 162–3 | Lung | 0·15 | 0·12 | 0·22 | 0·23 |
| 180 | Kidney | 0·55 | 0·50 | 0·55 | 0·70 |
| 181 | Bladder | 0·28 | 0·28 | 0·43 | 1·12 |
| 190–1 | Skin | 0·72 | 0·72 | 0·70 | 1·33 |
| 190 | Malignant melanoma | 1·20 | 1·13 | — | — |
| 191 | Other skin cancer | 0·68 | 0·53 | — | — |
| 193 | Brain | 0·78 | 0·65 | 0·64 | 0·87 |
| 194 | Thyroid | 2·33 | 1·93 | 2·84 | 16·50 |
| 195 | Suprarenal | — | 0·61 | — | — |
| 196 | Bone | 0·66 | 0·61 | 0·83 | 0·54 |
| 197 | Connective tissue | 0·83 | 0·71 | 0·86 | 0·62 |
| 200 | Lymphosarcoma, reticulosarcoma | 0·65 | 0·70 | — | — |
| 201 | Hodgkin's disease | 0·59 | 0·54 | 0·71 | 0·59 |
| 203 | Multiple myeloma | 0·72 | 0·79 | — | — |
| 204 | Leukaemia | 0·69 | 0·69 | 0·68 | 0·34 |
| 204·3 | Acute leukaemia | — | 0·73 | — | — |
| 204·1 | Myeloid leukaemia | — | 0·86 | — | — |
| 204·0 | Lymphatic leukaemia | — | 0·50 | — | — |
| 204·2 | Monocytic leukaemia | — | 0·65 | — | — |
| 202·5 | Other lymphoid tumours | 0·85 | 0·48 | — | — |

The majority of the individual tumour types reflect the general experience and the chance of a woman developing a tumour is between 40 and 70 per cent of that of a man doing so. Some tumours are even less common in the female than in the male. Cancer of the lip, larynx and lung and of the bladder fall into this category. The incidence, but not the mortality, of salivary gland tumours is equal in the two sexes; carcinoma of the hypopharynx kills equal proportions of men and women and carcinoma of the colon is of equal frequency in the two sexes. Malignant melanoma is appreciably commoner in women than in men and cancer of the thyroid is twice as frequent in women as in men.

### DISCUSSION

The greater frequency of tumours in males is well known but is usually passed over as a consequence of the different environments in which the two sexes live and work and the different internal, hormonal, chemical environments of the two sexes. The differences in tumour incidence in men and women follow similar patterns in different parts of the world (Ashley 1968) but the relative liability of men and women to develop tumours of particular sites varies widely; for example, carcinoma of the larynx is ten times as common in men as in women and thyroid cancer is twice as common in females as in males.

Some of these differences can be explained on anatomical and physiological grounds. The female reproductive system, the uterus and ovaries, undergoes a more or less regular cycle of growth and regression through the 30 or so years of active reproductive life in contrast to the testes and prostate of the male which function steadily, without much variation, until their activity declines as old age approaches. It is not therefore surprising that carcinogenesis is more frequent in the perpetually stimulated organs of the female than in those of the male. Similarly the breast is a rudimentary structure in the male, it contains few epithelial cells and undergoes no proliferation at puberty or during pregnancy. It is to be expected that tumour formation would be much rarer in such an organ than in the large active female mammary gland. The higher frequency of thyroid cancer in the female may also be related to the actions of endogenous hormones. Thyroid function, regulated by the hormones of the pituitary, is more readily deranged, either in the direction of excess or of deficit, in the female, and it is probable that this labile gland is more readily susceptible to loss of control of growth than is the more stable male thyroid.

Other differences may be attributed to environmental factors. Carcinoma of the lung is strongly associated with the habit of cigarette smoking (Kreyberg, 1962) which, in turn, is commoner in men than women. Similarly tumours of the lip and larynx occur in areas subjected more often to chemical insult in men than in women and in addition the anatomical changes in the male larynx at the time of puberty may play a part in tumour development in this site. Men are more likely than women to work in an industrial environment and are therefore more likely to be exposed to a variety of potentially carcinogenic substances. This factor may be relevant in the case of cancer of the lip, larynx and lung and also of the bladder where excretion products of industrial chemicals have been inculpated as contributory causes of bladder tumours (*British Medical Journal*, 1966).

In the remaining situations it is more difficult to explain the excess of cases in males and in particular to explain the occasional tumour, for example of the colon, in which the frequency in the two sexes is similar. Considerations of internal and external environment do not fully explain the differences because, as I have shown elsewhere (Ashley, 1968), the difference between the sexes exists at all ages and is present in the smallest of children whose environment, external and internal, does not differ in the two sexes. It is suggested that the reason for the differences in tumour incidence lies in a relatively greater efficiency of the immune mechanism of the female. One of the abnormal genes which has been identified as lying on the X chromosome is that for the congenital biochemical anomaly aggammaglobulinaemia (Garrie and Kendall, 1961) and therefore the normal gene, or one of them, for the production of serum gammaglobulin is located in this situation. Burch (1966) has shown that conditions of enhanced immunological activity, the auto immune diseases, are of more frequent occurrence in women than in men and also concludes that there are X chromosome genes concerned with immunity. If one of the body's defences against neoplasia is immunological in nature, if the earliest neoplastic cells may sometimes be recognised as " not self " and destroyed by a method analogous to that of graft rejection, the potentially better immunological capacity of the female could result in a lower frequency of tumours in this sex.

Such an hypothesis explains the female/male differential in tumour incidence but leaves two types of tumour which are relatively more frequent in the female than in the male. These are malignant melanoma and colonic cancer. In the case of malignant melanoma there is a possible connection with the pituitary gland. One of the hormones of the anterior pituitary is the " Melanocyte Stimulating Hormone " which in some circumstances produces an increase in skin pigmentation (McGuinness, 1963); if, in females, this function of the pituitary is also affected by the cyclical changes associated with the menstrual cycle it is also possible that cyclical changes in the activity of the epidermal melanocytes may make them more prone to neoplastic change in women than in men. This suggestion gains support from the occasional regression of malignant melanoma during pregnancy (Allen, 1955) and exacerbation at the menopause (Nathanson, Hall and Farber, 1967) although little benefit has so far been achieved in such patients by endocrine therapy or ablation (*Lancet*, 1961; Nathanson, Hall and Farber, 1967). It might be worth while considering further investigation of these lines of treatment in cases of this disease and studies of the hormonal status of groups of men and women who have developed malignant melanomas.

The problem posed by lesions of the colon and rectum is more more difficult. Colonic cancer occurs with equal frequency in men and women while rectal cancers, tumours of the distal end of the contiguous viscus occur in women at only about two thirds of the frequency with which they occur in men. An added complication is that there appears to be a racial difference as well. The female to male ratio for rectal cancer in the non-white component of the U.S. population is 1·06 while the ratio in the white population is 0·68 (Dorn and Cutler, 1955). This is a general difference. I calculated the weighted mean of the age adjusted incidence rates for the countries populated by peoples of African descent, Mozambique, Nigeria, Uganda, Jamaica and the Bantu people of South Africa and for the countries populated by people of North Western European stock from the data for " Cancer incidence for the five continents " (Doll, Payne and Waterhouse, 1966). The female to male ratio for colonic cancer was 1·44 in the African group and 1·03 in the European group: the ratio for rectal cancer was 1·13 for the African group and 0·63 for the European group. The reason for these differences is obscure. It is difficult to postulate a difference in endocrine sensitivity of the upper and lower parts of the large intestine present in Europeans but not in Africans. Similarly it is difficult to suggest environmental factors differing in their effect on the colons and rectums of people of different racial groups both in their home lands and in the urban environment of the ten Metropolitan centres studied by Dorn and Cutler (1955).

It is known that some cases of colonic cancer are consequent upon intestinal polyposis, a condition in which many thousands of glandular polyps are seen in the large intestine and which is inherited as an autosomal dominant condition (Veale, 1965). The proportion inherited in this way is, however, small, in my own experience 2 such cases, from one family, were encountered among 300 instances of cancer of the large intestine. It is possible that other genes located on the X chromosome are also concerned in the control of the growth of large intestine epithelium and that these are more frequent in people of one racial group than another. The problem posed by this curious distribution of tumours between the sexes is a difficult one and must be solved if a full understanding of the process of carcinogenesis is to be achieved.

SUMMARY

The frequencies of a number of tumours were compared in males and females after adjustment for the differing age structures of the populations of the two sexes. The data used were: estimates of tumour incidence in the five continents; the mortality experience of England and Wales; tumour incidences in 10 Metropolitan areas of the United States.

It was found that, apart from tumours of the reproductive organs which were commoner in women than in men, the chance of a female developing a tumour was between 40 and 70 per cent of that of a male. Exceptions to this finding were in the cases of tumours of the thyroid and malignant melanoma in which it was suggested that the cyclical endocrine activity of the female might be a contributory factor in the high frequency of these tumours in women and tumours of the lip, larynx, lung and bladder which are relatively more common in men and in which it was suggested that industrial environmental conditions and the habit of cigarette smoking were of aetiological importance.

It was suggested that there is a greater immunological potentiality in the female because of her two X chromosomes which are known to carry one at least of the genes concerned with the production of immunoglobulins. Anomalies in the sex distribution of colonic and rectal cancer were noted but were left unsolved.

This work was carried out with the aid of a research grant from the Welsh Hospital Board.

REFERENCES

ALLEN, E. P.—(1955) *Br. med. J.*, ii, 1067.

ASHLEY, D. J. B.—(1969) *Br. J. Cancer*, **23**, 21.

BRITISH MEDICAL JOURNAL—(1966) Report of Conference. *Br. med. J.*, ii, 761.

BURCH, P. R. J.—(1966) *J. theor. Biol.*, **12**, 397.

DOLL, R., PAYNE, P. AND WATERHOUSE, J.—(1966) ' Cancer Incidence in Five Continents '. U.I.C.C. Berlin (Springer-Verlag).

DORN, H. F. AND CUTLER, S. J.—(1955) Morbidity from Cancer in the United States. *Publ. Hlth Monogr.* No. 29. Washington D.C., U.S.A.

GARRIE, J. M. AND KENDALL, A. C.—(1961) *Br. med. J.*, i, 548.

KREYBERG, L.—(1962) ' Histological Lung Cancer Types '. Oslo (Norwegian Universities Press).

LANCET—(1961) Leading Article. *Lancet*, ii, 585.

McGUINESS, B. W.—(1963) *Ann. N.Y. Acad. Sci.*, **100**, 640.

NATHANSON, L., HALL, T. C. AND FARBER, S. (1967) *Cancer, N.Y.*, **20**, 650.

REGISTRAR GENERAL—(1960) Statistical Review of England and Wales for the year 1958. Part I. Tables Medical. London (H.M.S.O.).—(1961) Statistical Review of England and Wales for the year 1959. Part I. Tables Medical. London (H.M.S.O.).—(1962) Statistical Review of England and Wales for the year 1960. Part I. Tables Medical. London (H.M.S.O.).—(1963) Statistical Review of England and Wales for the year 1961. Part I. Tables Medical. London (H.M.S.O.).—(1964) Statistical Review of England and Wales for the year 1962. Part I. Tables Medical. London (H.M.S.O.).—(1965) Statistical Review of England and Wales for the year 1963. Part I. Tables Medical. London (H.M.S.O.).

VEALE, A. M. O.—(1965) ' Intestinal Polyposis '. *Eugen. Lab. Mem.* No. 40. Cambridge (Cambridge University Press).